# Management of mid-shaft clavicular fractures: comparison between non-operative treatment and plate fixation in 60 patients

**DOI:** 10.1007/s11751-016-0272-4

**Published:** 2017-01-04

**Authors:** B. M. Naveen, G. R. Joshi, B. Harikrishnan

**Affiliations:** Department of Orthopaedics, Armed Forces Medical College (AFMC), Pune, 411040 India

**Keywords:** Clavicle, Fracture, Mid-shaft, Plating

## Abstract

Clavicle fracture is a common injury due to its subcutaneous and relatively anterior position. Fractures affecting the middle third account for majority of all clavicular fractures. Both non-operative and surgical methods have been described for the management of this injury. However, there is no uniform consensus on the definite choice of treatment. Hence, this study was undertaken to compare conservative approach with primary internal plate fixation in mid-shaft clavicular fractures in terms of subjective outcome, functional outcome, the rates of nonunion and malunion and other local complications. Patients were allocated into two groups, each including 30 patients on alternate basis. Group 1 patients were managed conservatively, consisting of a figure-of-eight bandage and a sling, whereas patients of group 2 were treated surgically by plate fixation. Follow-up examination was done at 06 weeks, 03 and 06 months using patient’s subjective evaluation, functional outcome, radiographic assessment and other complications. The study showed that time to union was significantly shorter in patients treated surgically and this group also showed a favorable Constant shoulder score at all follow-ups. Though there was no statistically significant difference between the groups with regard to complication rate, subjective outcome or functional outcome, the surgical intervention group fared better especially when considering overall outcome results. The present study showed that the time to union was lesser, rate of malunion and nonunion was lower, and Constant shoulder scores were higher in the surgical group. This affirms that while conservative treatment remains the treatment of choice for simple undisplaced mid-shaft clavicle fractures, for displaced and comminuted fractures the surgical intervention gives better outcomes and early functional recovery in young active adults.

## Introduction

Clavicle fracture is one of the most common injuries around the shoulder girdle [1]. It has been reported that fractures of the clavicle account for approximately 2.6% of all fractures [2]. Incidence in males is usually highest in second and third decade which decreases thereafter as per age [3]. In females, it is usually bimodal, with peak incidence in young and elderly [4]. Allman [5] classified clavicle fractures into three groups based on their location along the bone. The middle-third fractures are most common and account for approximately 80–85% all clavicular fractures [6]. The narrow cross section of the bone in the middle shaft combined with typical muscle forces acting over it predispose to fracture the bone in this locality. Further, Robinson modified Allman classification based on the degree of displacement and comminution [3].

Most mid-shaft clavicle fractures generally unite with any method of immobilization. Hence, non-operative treatment was the established and accepted modality of these fractures. This was evident by extremely low nonunion rates shown by various studies done earlier [7, 8]. However, certain recent studies have shown suboptimal outcomes and a very high nonunion rates when displaced fractures are managed conservatively [9, 10]. Other shortcomings of non-operative treatment brought out were functional impairment of the shoulder and a non-cosmetic bump at the base of the neck possibly due to shortening of the clavicle and exuberant callus formation [9]. Restoration of normal length and alignment by surgical methods can prevent these drawbacks of conservative treatment. Good outcome with high union rates and low complication rates has been reported with various surgical modalities of primary fixation of the displaced fractures [11–14]. However, operative treatment has also got its own disadvantages such as surgical site infection, hypertrophic scar, hardware prominence and a repeat surgery for implant removal at times. Since mid-shaft clavicular fractures generally unite with most of the treatment modalities, clinical trials performed to compare these therapeutic options are rare. In addition, there is no uniform consensus yet on the definite choice of treatment for displaced mid-shaft clavicular fractures.

In the younger age group, apart from isolated clavicle fractures poly-traumatic injuries are also very common, and clavicular mid-shaft fracture remains a frequent entity. In such situations, the choice of treatment remains a constant dilemma for achieving maximum pre-fracture functional status. Hence, in this study we endeavored to find an evidence-based answer to select the better approach for the management of acute displaced mid-shaft clavicular fractures. The aim of this study was to compare sixty patients with mid-shaft clavicular fractures treated either by conservative approach or primary internal plate fixation in terms of functional outcome, the rate of nonunion, malunion and overall local complications up to 6 months after treatment. In addition, it was also intended to study the clinical response in terms of subjective outcome and the advantages and disadvantages of both the treatment modalities.

## Materials and methods

A comparative study of management of mid-shaft clavicle fractures (Robinson type 2b) was carried out at a tertiary care teaching hospital between Jun 2011 and Jun 2013. Study population included patients in age group of 20 and 50 years with completely displaced fracture of the mid-shaft clavicle. Patients with severe brain injury, intubated patients, those with open fractures or ipsilateral limb fracture and those with injury precluding operative fixation within 7 days of admission were excluded from the study.

It is a non-randomized comparative trial with equal allocation, consisting of 60 patients with freshly diagnosed mid-shaft clavicular fractures. Group 1 consisted 30 patients who were managed conservatively and group 2 had 30 patients who were treated surgically. Patients were allocated into both the treatment groups on alternate basis, i.e., group 1 followed by group 2 (Table 1).

**Table 1:**
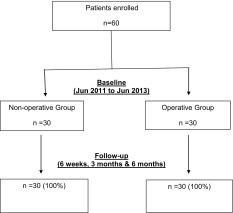
Flowchart representation of patient recruitment and the follow-up rates

In the outpatient department of the hospital, the surgeon or orthopedic resident identified the patients eligible for the study and the study protocol was instituted. Patients were informed in detail by the treating surgeon regarding the advantages and disadvantages of both operative and non-operative care. The nature of the study was explained to all the patients in their own language that they understand and necessary consent was obtained after the patients gave their willingness to participate in the study.

**Fig. 1:**
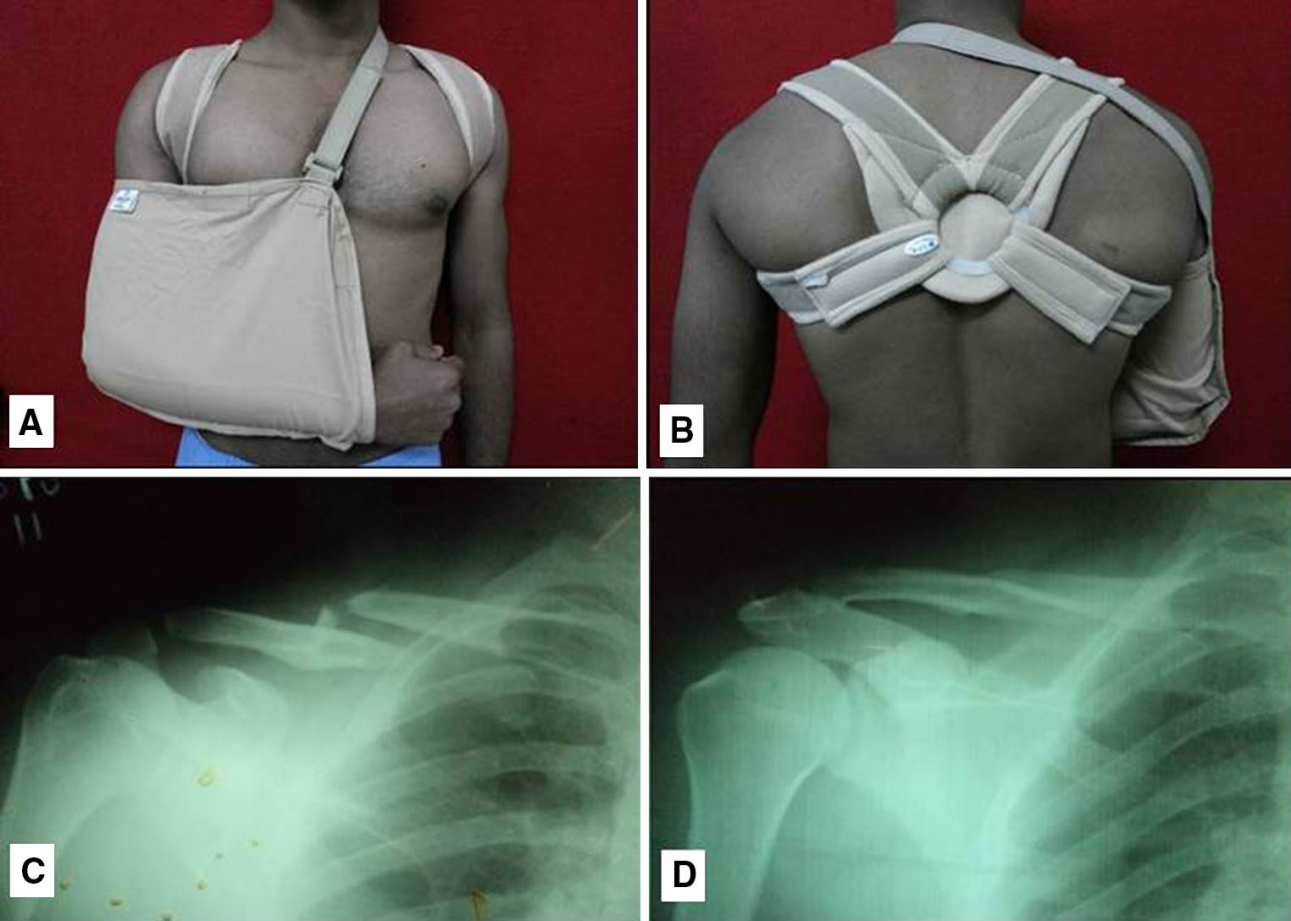
**a** Figure-of-eight bandage with shoulder arm pouch-anterior view. **b** Figure-of-eight bandage with shoulder arm pouch-posterior view. **c** Initial radiograph of the fracture at presentation. **d** Fracture union after 6 months of conservative treatment

**Fig. 2:**
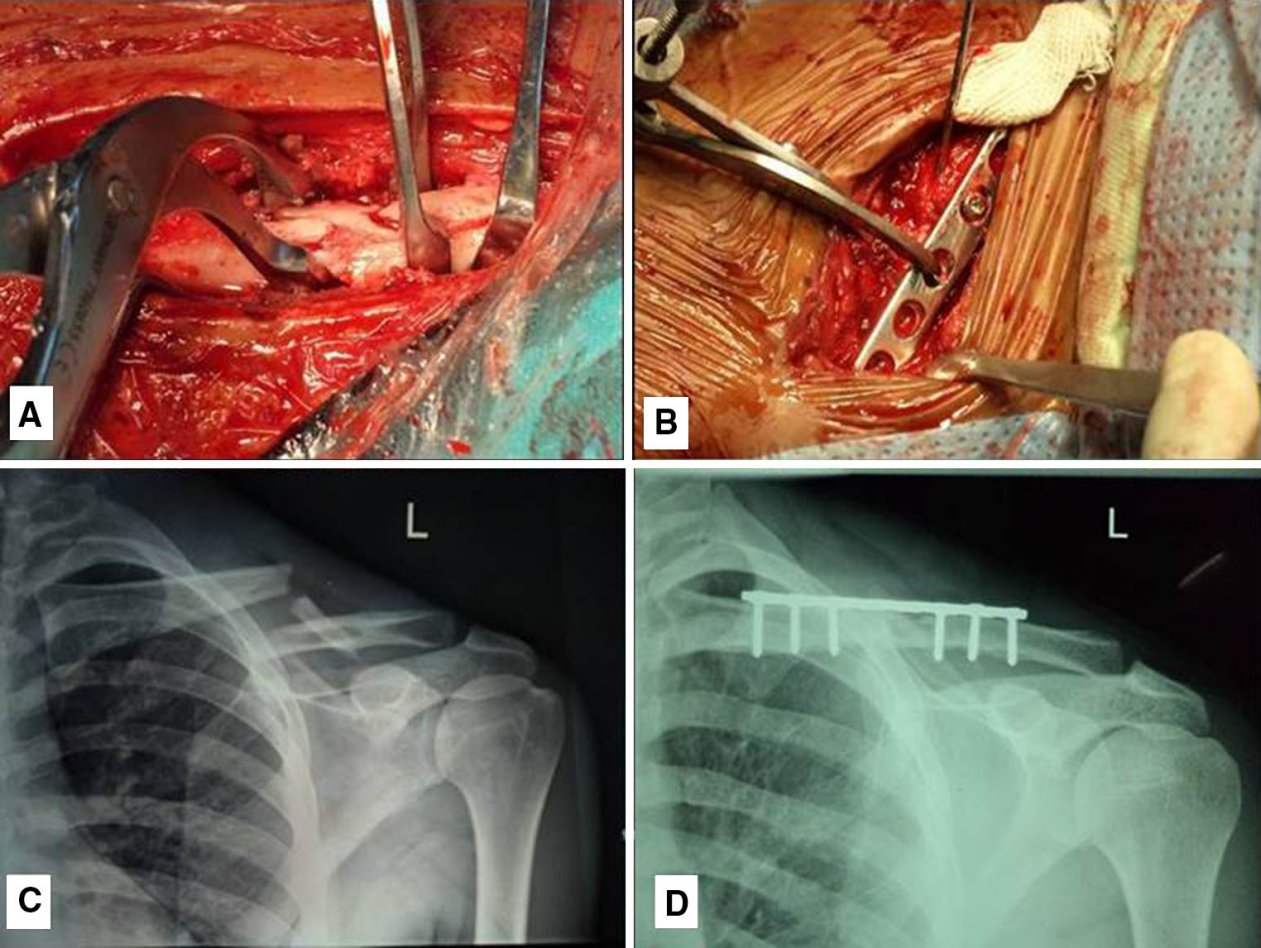
**a** Intra-operative fracture reduction. **b** Fracture fixation with 3.5 mm DCP. **c** Radiograph before fracture fixation. **d** Fracture union after 6 months of surgical treatment

Group 1 patients were managed conservatively, consisting of a figure-of-eight bandage (Fig. 1a–d) and a sling, whereas patients of group 2 were treated surgically by plate osteosynthesis (Fig. 2a–d). Patients allocated to plate fixation group underwent the operation within seven days after the injury. An 8–10 cm skin incision was placed on the line joining sternal notch to anterior edge of acromion centered over fracture site on the affected side. Platysma was released from lateral side and supraclavicular nerves protected wherever possible. Subsequently the clavipectoral fascia was incised and elevated. Fractures fragments identified and reduced under vision. The plate (3.5 mm DCP) was contoured and applied over the superior aspect of the clavicle taking care not to injure the underlying neurovascular structures. Comminuted fragments secured with lag screws wherever possible.

A rehabilitation protocol was started after removal of the bandage in group 1 and immediately after plate fixation in group 2. Gentle pendulum exercises of the shoulder in the sling/arm pouch were allowed as per pain tolerance immediately after surgery in surgical treated group and after 3 weeks in conservative group. At 3 weeks, gentle active range of motion of the shoulder was allowed with abduction limiting to 90°. Subsequently, active range of motion exercises that are to be performed at home is advised. At four to 6 weeks, active to active assisted range of motion in all planes was allowed. When fracture union (defined as radiographic union with no pain or motion with manual stressing of the fracture) was evident, muscle strengthening exercises were also allowed. At eight to 12 weeks, isometric and isotonic exercises were prescribed to the shoulder girdle muscles with a return to full activities (including sports) at 3 months.

Regular follow-up was done every fortnight for initial 6 weeks, then at 06 weeks, 03 and 06 months using patient’s subjective evaluation, functional outcome and radiographic assessment. Patients’ subjective evaluation was investigated by direct interview at the follow-up visits. Functional outcome was graded on the standardized clinical evaluation and completion of the Constant and Murley score [15]. Fracture healing was monitored by periodic radiographic examinations on two planes. The fracture was considered to be united when there was no tenderness at the fracture site with full function of the limb clinically and when the bridging callus was seen radiologically. Both the clinical and radiologic unions were assessed by an independent surgeon. An adverse event or complication was defined as any event that necessitated another operative procedure or additional medical treatment.

## Statistics

The data analysis was done using SPSS software version 17. We have used Fisher’s exact test, Chi-square test and 2 independent sample t-tests to find the association/significance between group 1 and group 2. The observed results were determined to be significant if the *P* value was <0.05 and not significant if it was >0.05.

The institute’s ethics committee approval was taken before the commencement of study.

## Results

There was no statistically significant difference between the group 1 and group 2 with regard to demographic parameters such as mode of injury, age and sex of patients, side affected, presence of associated injuries and type of fracture as per Robinson’s classification (Table 2).

**Table 2:** Patient demographics and *P* value between the two groups

Demographic parameters	Group 1	Group 2	*P* value (<0.05 taken as significant)
**Age (mean)**	35.20	32.43	0.219
**Sex**			
Male	27	26	0.999
Female	3	4	
**Mode of injury**			
RTA	20	19	0.999
Fall	7	7	
Sports injury	3	4	
**Side affected**			
Dominant	13	12	0.999
Non-dominant	17	18	
**Presence of associated injuries**			
Present	6	8	0.542
Absent	24	22	
**Robinsons classification**			
2B1	10	15	0.295
2B2	20	15	

**Fig. 3:**
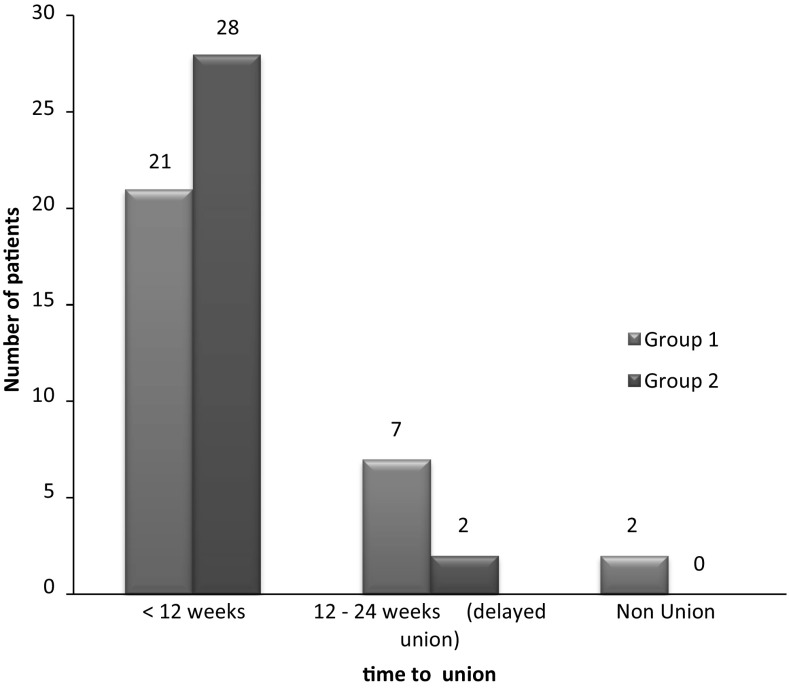
Time to union with respect to treatment group

**Fig. 4:**
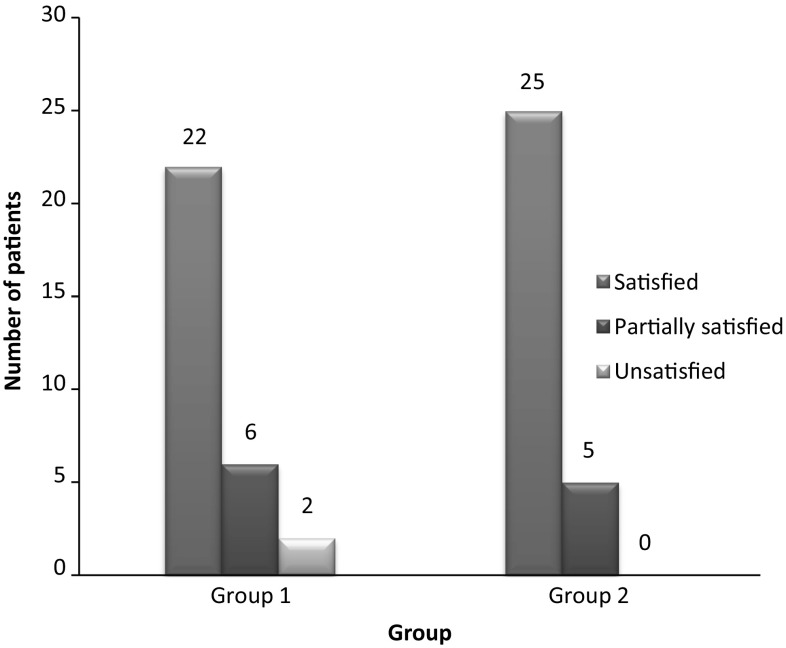
Subjective evaluation at six months follow-up

The time to union was significantly shorter (*P* < 0.05) in patients treated surgically (Fig. 3). The fracture united in 93% of the patients in group 1, whereas all patients had fracture union in group 2. Fracture union was early and seen in more number of patients in group 2 as compared to group 1. Around 73% of patients were fully satisfied, with the treatment at the end of 6 months in group 1, as compared to 83% in group 2 with the treatment (Fig. 4).

The mean Constant score was higher in the surgically treated group in comparison with conservatively managed group at the end of 6 weeks, 3 and 6 months, and it was statistically significant (Table 3).

**Table 3:** Comparison of Constant shoulder score between the groups at 6 weeks, 3 and 6 months

Constant score at	Group 1		Group 2		*P* value
	Mean	SD	Mean	SD	
Sixth week	63.87	5.75	71.80	4.87	<0.001
Third month	75.77	5.96	83.63	4.82	<0.001
Sixth month	89.60	6.64	94.00	2.99	0.001

Nine patients (30%) in group 1 had various complications such as malunion with cosmetic deformity, nonunion and restriction of shoulder movements, as compared to 6 patients (20%) in group 2 who had scar-related problems and hardware prominence along with the one malunion (Table 4). Malunion and nonunion rates were higher in conservative group in comparison with the surgical group. However, complications of surgical group were generally related to surgical technique and the implant. Overall, the complication rate in the conservative group was relatively higher.

**Table 4:** Various complications in both the groups and their *P* value

	Treatment group		Total	*P* value
	Group 1	Group 2		
Malunion with cosmetic deformity	6	1	7	0.103
Nonunion	2	0	2	0.492
Scar problems	0	3	3	0.237
Hardware prominence	0	2	2	0.492
Restriction of ROM	1	0	1	0.999
Total	9 (30%)	6 (20%)	15 (25%)	0.371

## Discussion

In the past, conservative management was the mainstay of treatment for all clavicle fractures in middle third irrespective of displacement and comminution as clavicle has excellent power of remodeling. Conservative treatment with figure-of-8 bandage aligns the displaced fragments in an acceptable manner and results in a good functional outcome. However, a recent meta-analysis revealed higher nonunion rates for displaced fractures treated non-operatively (15%) than operatively (2.2%) with modern internal fixation techniques [10]. Multiple recent trials have also revealed higher incidence of residual pain, nonunion, malunion, shoulder weakness, decreased shoulder endurance, inferior patient and surgeon-oriented outcome scores, and lower overall satisfaction after non-operative management of mid-shaft clavicle fractures [12, 16]. The operative management of these fractures with plating or nailing was reserved only for a subset of population with open fractures or highly displaced fractures.

The existing literature reports two sets of incidence of these fractures: The first is the largest and is associated with young active population (sports, motor vehicle accidents), whereas the second is associated with elderly individuals (osteoporotic fractures with simple falls) [4]. A direct blow to the shoulder is the most common mechanism of injury that produces a mid-shaft fracture of the clavicle. As the shoulder is subjected to a high compression force from lateral side, the clavicle and its articulations are the main areas to get affected as they resist these forces. Most (85%) clavicle fractures occur in the mid-shaft as the bone is narrowest and enveloping soft tissue structures (which may help dissipate injury force) are most scarce [17]. In our study, the age group was 20–50 years. The mean age was 35.2 years in group 1 and 32.4 years in group 2. The dominant side was affected in 25 cases (41.66%) out of 60 subjects, whereas remaining 35 cases (58.34%) had fracture on the non-dominant side which similar to the incidence reported in the literature [18, 19]. Functional impairment of the shoulder and the upper limb can be extremely variable. A careful clinico-radiologic assessment is absolutely necessary to exclude associated chest injuries, such as pneumothorax or haemothorax, which are reported in the literature to occur at rates of up to 3% [8]. In the present study, 14 patients (23.3%) had associated injuries. However, none of these patients had pneumothorax or haemothorax or neurovascular injury.

Generally, the clavicle fractures undergo operative fixation within first 10–14 days from the time of injury. However, various studies report increased number of complications, if the primary fixation is delayed for more than 2 weeks [20]. All patients underwent surgery within first 7 days in our study which might have contributed to higher rates of bony union. The advantages of plate fixation include immediate rigid stabilization and pain relief and it also facilitates early mobilization. The rehab protocol instituted in both the treatment groups has been discussed in the previous section. The early mobilization in the surgical group helped the patients to maintain their shoulder strength and early shoulder function, whereas conservatively treated patients had their shoulder immobilized for 3 weeks, which might have resulted in shoulder weakness and delayed shoulder function. Hence, the functional outcome as measured by Constant shoulder score was higher in surgically treated patients at all follow-ups in comparison with non-surgical group. Moreover, the earlier rehabilitation might have contributed to higher rates of bony union and early functional recovery as evident from our results.

The average duration required for union in conservative group was 11.29 weeks, as compared to 9.27 weeks in operative group. There is a statistically significant difference in the mean duration to union in both the groups similar to other studies [20, 21]. Majority of the patients in conservative group returned to their pre-injury activity levels by around 16 weeks, whereas in the surgical group it was around 12 weeks.

Previous studies in adults have shown a higher rate of patient satisfaction after non-operative treatment of clavicle fractures [16, 22]. But, patient-reported satisfaction scores may be superior with an early surgical stabilization in some circumstances. A multicenter trial reported better functional outcomes, lower malunion and nonunion rates, and a shorter overall time to union in operatively treated clavicle fractures after plate fixation [12]. In our study, the mean Constant shoulder score for group 1 was 63.87, 75.77 and 89.60 at 6 weeks, 3 and 6 months, respectively. However, for group 2, it was 71.80, 83.63 and 94.00 at 6 weeks, 3 and 6 months, respectively. There was a difference of 7.93 points in favor of surgical group at 6 weeks, 7.86 points at 3 months and 4.40 points at 6 months. At the end of 6 months, 93.33% patients achieved an excellent result (Constant score >90) in the surgically treated group as compared to 80% in the conservative group. 6.66% of the patients had a good score in surgical group (Constant score between 70 and 90) as compared to 13.33% in the conservative group. 6.66% patients had poor score in the conservative group (Constant score <70) as compared to none in the surgical group.

Earlier trials have analyzed the risk of shoulder dysfunction after conservative treatment, which generally was attributed to shortening of the bone segment, residual bone deformity, loss of force and persistent pain [23]. Some studies have observed lesser number of consolidation defects after surgical fixation as compared to conservative treatment, whereas others have demonstrated a 37% risk of adverse events after a surgical procedure possibly due to invasion of the periosteal structures that can lead to nerve damage, blood loss and post-traumatic hematoma, which can delay fracture healing [19].

In our study, we had a total of 15 patients (25%) out of 60 with complications across both groups. Out of 15 patients with complications, 9 patients (30%) belonged to non-surgical group and 6 patients (20%) belonged to surgical group. Though the difference was not significant when total number of complications was taken into account in both the groups, symptomatic malunion and nonunion was more common in conservative group than the surgical group. There were no surgical site infection, complex regional pain syndrome or neurovascular problems in any of our subjects. The study results are in line with more dated reports of outcomes of operative treatment of displaced mid-shaft clavicular fractures that show a complication rate of 23% and more. Some trials indicate that although clavicular deformities are complex and hard to analyze, shortening by 1.5–2 cm may result in an increased incidence of clinical symptoms. Shortening is one parameter which can be measured [23]. In the present study, there were six patients (20%) with symptomatic malunion with a cosmetic deformity in conservative group as compared to one patient (3.33%) in the surgical group. This patient in the surgical group had premature loading of the injured extremity because of which the plate got bent and resulted in malunion.

Several recent studies have shown high union rates with surgical management using a variety of internal fixation devices, including plating and IM pin or rod fixation [11]. In addition, there is also strong evidence that the nonunion rate after conservative treatment may be higher than previously reported, particularly in certain patients and fracture types. In this study, we had 2 nonunions (6.66%) out of 30 patients in conservative group as compared to none in surgical group. These two patients with nonunion underwent operative treatment at a later date. Our results with regard to various complications compare well with the existing literature and the published studies on the subject.

Our study has few strengths and limitations. Though the sample size is small and was not calculated prior to the study, the study has the sufficient power (>90%) to identify a standardized effect size in the Constant score of 0.5 at the final follow-up. It is a prospective non-randomized comparative trial, wherein there was no selection bias and the baseline demographic characteristics of the subjects in both the groups were almost similar, which reduced the chance of any other bias in the outcome. However, certain residual confounding factors in the results cannot be excluded as only a few were considered. The major strength of the study was the 100% follow-up in both the groups, though it was only 6 months.

From our study, we have noticed that in the surgical group, time to union was shorter with almost 100% union rates. More patients were satisfied and subjective outcome was better. The Constant shoulder scores were also significantly higher at all follow-ups. The numbers of complications were lesser and many of them were implant related and surgical technique related. On the other side, patients treated conservatively took longer time to unite and had more number of malunions and nonunions. Subjective outcome was inferior as compared to surgical group, and Constant shoulder scores were also lower at all follow-ups. Hence, in a young, active patient, surgical fixation of an acute displaced mid-shaft clavicle fracture in the form of plating appears to result in improved outcome. Plate fixation in these individuals is a reasonable option to maintain anatomic reduction and achieve union with restoration of maximal shoulder function.

The limited complications of surgical group seen in the present study were implant and surgical technique related and can be minimized with better availability of modern implants and good surgical technique. Recently, with the advent of pre-contoured locking plates, the incidence of hardware prominence has decreased. These plates are particularly beneficial in osteoporotic and severely comminuted fractures. The usage of pre-contoured anatomic clavicle plates and an anteroinferior approach for the fixation may minimize many of these complications. The conservative treatment remains the gold standard in treatment of simple undisplaced mid-shaft clavicle fractures, but for displaced and comminuted fractures surgical intervention is appropriate especially in young active adults. If implants and expertise is available, with a good surgical technique operative treatment might give satisfactory and superior results over nonoperative treatment. Although certain multicenter trials support the use of primary operative fixation for diaphyseal fractures [12], the quantum of this treatment effect on the outcome may not be sufficient enough to justify a surgical treatment to all patients.

In conclusion, anatomic reduction with plate fixation and early mobilization of displaced clavicle fractures is a viable treatment option, especially in young active adults with good outcomes and no major complications. There is also a need for further large multicenter prospective randomized controlled trials in order to generalize this preference of operative fixation over non-operative management in acute displaced mid-shaft clavicular fractures for all patients.
